# Why Babies die in the first 7 days after birth in Somalia Region of Ethiopia?

**DOI:** 10.1097/MS9.0000000000000690

**Published:** 2023-04-18

**Authors:** Gizachew G. Mekebo, Gudeta Aga, Ketema B. Gondol, Belema H. Regesa, Birhanu Woldeyohannes, Temesgen S. Wolde, Getachew Tadesse, Agassa Galdassa, Kebede L. Adebe, Hirko Ketema

**Affiliations:** a College of Natural and Computational Science, Ambo University, Ambo; b Addis Ababa Transport Bureau, Addis Ababa, Ethiopia

**Keywords:** determinants, early neonatal death, Somalia region of Ethiopia, the first 7 days of life after birth

## Abstract

Early neonatal death, which occurs during the prenatal period, is defined as the death of a newborn between 0 and 7 days after birth. It is one of the major public health problems in several developing countries. This study aimed to determine the early neonatal mortality rate and identify determinants of early neonatal mortality in Somalia region of Ethiopia.

**Methods:**

The data used for this study were drawn from the 2019 Ethiopia Mini Demographic and Health Survey (2019 EMDHS) data. A multivariable logistic regression model was used to identify the determinants of early neonatal mortality. Adjusted odds ratio (AOR) with 95% CI was used to examine the association of factors with early neonatal mortality.

**Results:**

A total of 637 live births were included in this study. The early neonatal mortality rate in the study was 44 (95% CI: 31, 65) deaths per 1000 live births. Male babies (AOR: 1.628; 95% CI: 1.152–4.895), babies delivered at home (AOR: 2.288; 95% CI: 1.194–6.593), and babies born from uneducated mothers (AOR: 2.130; 95% CI: 1.744–6.100) were at increased risk of death in the first 7 days of life after birth. Contrarily, decreased risk of death of babies in the first 7 days of life after birth was associated with urban residence (AOR: 0.669; 95% CI: 0.033–0.721) and singleton (AOR: 0.345; 95% CI: 0.070–0.609).

**Conclusion:**

The early neonatal mortality rate in the region was high. The study revealed that sex of child, place of residence, birth type, mother’s educational level, and place of delivery were the determinants of the death of babies in the first 7 days of life after birth. Hence, providing health education to uneducated mothers and enhancing institutional delivery is recommended to minimize the early neonatal mortality rate in the region.

## Introduction

Highlights

- This study was an attempt to determine the early neonatal mortality rate and identify determinants of early neonatal mortality in Somalia region of Ethiopia using 2019 Ethiopia Mini Demographic and Health Survey data (2019 EMDHS).
- The early neonatal mortality rate in the study area was 44 (95% CI: 31, 65) deaths per 1000 live births.
- Sex of the child, residence, birth type, mother’s educational level, and place of delivery were determinants of the death of babies in the first 7 days of life after birth.
- Providing health education to uneducated mothers and enhancing institutional delivery is recommended to minimize the early neonatal mortality rate in the region.

Early neonatal death, which occurs during the prenatal period, is defined as the death of a baby between 0 and 7 days after birth^1^. Early neonatal death is one of the major public health problems in several developing countries and has economic, social, and health implications for families in particular and societies at large^2^. It accounts for about 73% of all postnatal deaths globally^1^.

Neonatal death is significantly influenced by perinatal mortality, which comprises stillbirths and early neonatal deaths. Worldwide, perinatal mortality accounts for about three-fourths of neonatal deaths, and the day of birth is the most perilous moment for newborns^3^. Globally, the neonatal death rate has fallen by 41%, from 31 per 1000 live births in 2000 to 18 in 2017, which is a smaller reduction in mortality compared with the 54% reduction in mortality for under-five children^4^. The WHO reports that Sub-Saharan Africa is among the regions with the highest neonatal mortality rate^5^.

In Ethiopia, neonatal mortality, death in the first month after birth, increased from 29 deaths per 1000 live births in 2016 to 33 deaths per 1000 births in 2019^6^. The Ethiopia Demographic and Health Survey reports also show that the neonatal mortality rate in Somalia region of Ethiopia has increased from 41 deaths per 1000 live births in 2016^7^ to 45 deaths per 1000 births in 2019^6^. Moreover, Somalia region of Ethiopia is the region with the highest death rate of babies in the prenatal period in the country in 2016, with a rate of 50 deaths per 1000 live births compared to the national average of 33 deaths per 1000 live births^7^.

Place of residence, sex of the child, antenatal care, birth type, having a skilled attendant at birth, previous history of perinatal mortality, obstetric complication during labor, birth weight, maternal educational level, father’s educational level, history of previous abortion, preceding birth interval, gestational age, wealth index of family, birth order, maternal anemia, maternal HIV, maternal employment, marital status, parity, age of mother, family size, place of delivery, mode of delivery, maternal death at birth, and late breastfeeding initiation were factors reported to be significant determinants of early neonatal mortality by several studies^8^–^43^.

In spite of the efforts that have been made in Ethiopia to reduce the death rate of babies in the perinatal period, it is still high in some regions of Ethiopia^44^, including Somalia region with the highest prenatal mortality in the country^8^. Despite the high death rate of babies in the perinatal period in Somalia region of Ethiopia, no adequate studies have been conducted to identify what factors induce the death of babies in the perinatal period in the region, particularly using the national representative data. Many researches need to be done to plan and implement the appropriate strategies that help in minimizing early neonatal mortality, which accounts for about 73% of postnatal mortality. The findings of this research will provide information regarding factors associated with the death of babies in the first 7 days of life. Thus, this study aimed to determine the early neonatal mortality rate and identify the determinants of early neonatal mortality in Somalia region of Ethiopia.

## Methods

### Data source

The data used for this study were drawn from the 2019 Ethiopia Mini Demographic and Health Survey data (2019 EMDHS). The 2019 EMDHS is the second EMDHS to be implemented in Ethiopia, and the data were collected from 21 March to 28 June 2019^7^. This study used the Kids data belonging to the Somalia region from the 2019 EMDHS data. Somalia region of Ethiopia is one of the administrative regions in the country.

### Study variables

The outcome variable was the death of a baby within the first 7 days of life after birth. It is coded as 1 if the baby died in the first 7 days of life after birth and 0 otherwise. Independent variables were sex of child, place of residence, birth type, birth order, mode of delivery, place of delivery, age of mother, maternal educational level, marital status, wealth index, and family size.

### Statistical data analysis

The data analysis was done using SPSS version 26. Frequencies and percentages were used to describe the background characteristics of the participants of the study. A bivariate analysis was done to select the independent variables to be included in the multivariable logistic regression analysis. The multivariable logistic regression analysis was done to identify the determinants of early neonatal mortality. The statistical significances of the independent variables were declared at *P* value less than 0.05. Adjusted odds ratio (AOR) with 95% CI was used to examine the association between the independent variables and early neonatal mortality. The Hosmer and Lemeshow test was used to check the goodness of fit of the model. The model, with a Hosmer and Lemeshow test significance value of 0.319, was found to be a good fit.

## Results

### Demographic and socioeconomic characteristics of the study participants in Somalia region of Ethiopia, 2019 EMDHS

A total of 637 live births, of which 28 died in the first 7 days of life after birth, were included in this study. Of the total live births, 356 (55.9%) were males, 527 (82.7%) were from urban, 615 (96.5%) were singletons, 393 (61.7%) had birth order of fourth and above, 632 (99.2%) were born vaginally, and 517 (81.2%) were born at home. With regard to mother’s level of education, age of mother, family size, marital status, and wealth index of the household, 538 (84.5%) were uneducated, 543 (85.2%) were in the age range of less than 25 years, 529 (83.0%) were from families of size 5 and above, 608 (95.4%) were married/living together with partners, and 545 (85.6%) were from poor families.

### Early neonatal mortality rate in Somalia region of Ethiopia, 2019 EMDHS

The early neonatal mortality rate was 44 (95% CI: 31, 65) deaths per 1000 births. The early neonatal mortality rate was higher among male babies (51 deaths per 1000 live births), rural babies (46 deaths per 1000 live births), multiple births (182 deaths per 1000 live births), first-born babies (90 deaths per 1000 live births), home deliveries (46 deaths per 1000 live births), babies born to mothers aged 35 years and above (66 deaths per 1000 live births), and babies born in poor families (46 deaths per 1000 live births). In the bivariate analysis, sex of child, place of residence, birth type, place of delivery, maternal educational level, age of mother and family size had an association with early neonatal mortality at 5% level of significance. These variables were included in the multivariable analysis (Table 1).

**Table 1:** Demographic and socioeconomic characteristics of study participants and early neonatal mortality rate in Somalia region of Ethiopia, 2019 EMDHS

			Child status within the first 7 days of life after birth			
Variables	Categories	Frequency (%)	Died (*n*, %)	Alive (*n*, %)	Early neonatal mortality rate per 1000 live births	*P*
Sex of child	Male	356 (55.9)	18 (5.1)	338 (94.9)	51	0.017*
	Female	281 (44.1)	10 (3.6)	271 (96.4)	36	
Place of residence	Urban	110 (17.3)	4 (3.6)	106 (96.4)	36	0.004*
	Rural	527 (82.7)	24 (4.6)	503 (95.4)	46	
Birth type	Single	615 (96.5)	24 (3.9)	591 (96.1)	39	0.019*
	Multiple	22 (3.5)	4 (18.2)	18 (81.8)	182	
Birth order	1st	78 (12.2)	7 (9.0)	71 (91.0)	90	0.157
	2nd–3rd	166 (26.1)	4 (2.4)	162 (97.6)	24	
	4th and above	393 (61.7)	17 (4.3)	376 (95.7)	43	
Mode of delivery	Vaginal	632 (99.2)	27 (4.3)	605 (95.7)	43	0.143
	Cesarean	5 (0.8)	1 (20.0)	4 (80.0)	200	
Place of delivery	Home	517 (81.2)	24 (4.6)	493 (95.4)	46	0.002*
	Health institution	120 (18.8)	4 (3.3)	116 (96.7)	33	
Maternal educational level	No education	538 (84.5)	25 (4.6)	513 (94.2)	46	0.033*
	Primary	82 (12.9)	2 (2.4)	80 (97.6)	24	
	Secondary and higher	17 (2.7)	1 (5.9)	16 (94.1)	59	
Age of mother	Less than 25	543 (85.2)	22 (4.1)	521 (95.9)	41	0.048*
	25–34	33 (5.2)	2 (6.1)	31 (93.9)	61	
	35+	61 (9.6)	4 (6.6)	57 (93.4)	66	
Family size	Less than 5	108 (17.0)	7 (6.5)	103 (93.5)	65	0.039*
	5 and above	529 (83.0)	21 (4.0)	508 (96.0)	40	
Marital status	Married/living together	608 (95.4)	25 (4.1)	583 (95.9)	41	0.470
	Widowed	10 (1.6)	1 (10.0)	9 (90.0)	100	
	Divorced/no longer living together/separate	19 (3.0)	2 (10.5)	17 (89.5)	105	
Wealth index	Poor	545 (85.6)	25 (4.6)	520 (95.4)	460	0.892
	Medium	27 (4.2)	1 (3.7)	26 (96.3)	37	
	Rich	65 (10.2)	2 (3.1)	63 (96.9)	31	

* Significant at 5% level of significance.

2019 EMDHS, 2019 Ethiopia Mini Demographic and Health Survey.

### Determinants of early neonatal mortality in Somalia region of Ethiopia

Multivariable logistic regression analysis revealed that sex of child, place of residence, birth type of child, maternal educational level, and place of delivery were factors significantly determining the death of babies in the first 7 days of life (Table 2).

**Table 2:** Determinants of early neonatal mortality in Somalia region of Ethiopia, 2019 EMDHS

Variables	Categories	AOR (95% CI of AOR)	*P*
Sex of child	Male	1.628 (1.152–4.895)	0.031*
	Female	1	
Place of residence	Urban	0.669 (0.033–0.721)	0.000*
	Rural	1	
Birth type	Single	0.345 (0.070–0.609)	0.041*
	Multiple	1	
Age of mother	Less than 25	0.519 (0.180–1.495)	0.224
	25–34	1.336 (0.284–6.276)	0.714
	35+	1	
Place of delivery	Home	2.288 (1.194–6.593)	0.035*
	Health institution	1	
Maternal educational level	No education	2.130 (1.744–6.100)	0.015*
	Primary education	1.403 (0.128–3.274)	0.122
	Secondary and higher	1	
Family size	Less than 5	0.589 (0.226–1.797)	0.152
	5 and above	1	

* Significant at 5% level of significance.

AOR, adjusted odds ratio; 2019 EMDHS, 2019 Ethiopia Mini Demographic and Health Survey.

The odds of dying in the first 7 days of life after birth among the male babies was 1.628 (AOR: 1.628; 95% CI: 1.152–4.895) times higher compared to female babies. The odds of the death of babies in the first 7 days of life after birth among urban residences was 0.669 (AOR: 0.669; 95% CI: 0.033–0.721) times lower compared to rural residences. The odds of the death of babies in the first 7 days of life after birth among single births was 0.345 (AOR: 0.345; 95% CI: 0.070–0.609) times lower compared to multiple births. The odds of the death of babies in the first 7 days of life after birth among those born at home was 2.288 (AOR: 2.288; 95% CI: 1.194–6.593) times higher compared to those born at a health institution. Moreover, the odds of the death of babies in the first 7 days of life after birth among babies born from uneducated mothers was 2.130 (AOR: 2.130; 95% CI: 1.744–6.100) times higher compared to babies born from mothers with secondary and higher education (Table 2).

## Discussion

This study was an attempt to determine the early neonatal mortality rate and identify the determinants of early neonatal mortality in Somalia region of Ethiopia. A total of 637 live births were included in the study. Of the total live births, 28 (4.4%) died within the first week of life. The early neonatal mortality rate was 44 deaths per 1000 births (95% CI: 31, 65).

This study found that babies born from mothers living in urban areas were less likely to die within the first 7 days of life after birth compared to babies born from mothers living in rural areas. This agrees with studies^9^,^10^,^12^,^14^,^15^. This might be due to the fact that mothers living in urban areas have better education and awareness about nutrition during pregnancy. Male babies were more likely to die within the first 7 days of life after birth compared to female babies. This result is consistent with the previous studies^12^,^14^,^17^,^19^–^21^,^23^. The possible reason could be that males are biologically weaker than females. Babies of single births were less likely to die within the first 7 days of life after birth compared to babies of multiple births. This result is in line with the studies^14^,^17^,^25^,^26^,^28^. This could be because singletons are less likely to be born with low birth weight than multiple births, which may result in a risk of death.

This study also found that the babies born at home were more likely to die within the first 7 days of life after birth compared to babies born at health institutions. This result is in line with the studies^14^,^15^,^21^,^25^. This might be because babies born at home are suspected of being in danger and less care is given during delivery compared to babies born at health institutions. Babies born from uneducated mothers were more likely to die within the first 7 days of life after birth compared to babies born from mothers with secondary and higher education. This agrees with the studies^14^,^21^,^26^,^29^,^31^,^32^. The possible justification could be that uneducated mothers are less aware of the danger signs of pregnancy and may also be poor at nutrition during pregnancy compared to educated mothers.

### Strength and limitation of the study

The strength of the study was that it used national representative data. The main limitation of this study was that some important variables such as weight at birth, ANC follow-up, gestational age, breastfeeding, preceding birth interval, HIV status of the mother, and anemia status of mother, which could have an association with early neonatal mortality, were not included in the study because of unavailability or high missing values in the dataset we used for the analysis.

## Conclusion

The aim of this study was to determine the early neonatal mortality rate and identify the determinants of early neonatal mortality in Somalia region of Ethiopia. The study revealed that the early neonatal mortality rate in Somalia region of Ethiopia was 44 (95% CI: 31, 65) deaths per 1000 births. The study revealed that factors such as sex of child, place of residence, birth type, mother’s educational level, and place of delivery determine the death of babies in the first 7 days of life after birth. Hence, providing health education to uneducated mothers and enhancing institutional delivery is recommended to minimize the early neonatal mortality rate in the region.

## Ethical approval

The data used for this were secondary data and are publicly available and have no personal identifiers.

## Consent

Not applicable.

## Sources of funding

This study was not funded.

## Author contribution

G.G.M.: conceptualization, data curation, formal analysis, investigation, methodology, software, validation, and writing – original draft; G.A., K.B.G., B.H.R., B.W., T.S.W., G.T., A.G., K.L.A., and H.K.: formal analysis, methodology, software, and writing – review and editing.

## Conflicts of interest disclosure

The authors declare that there are no conflicts of interest.

## Research registration unique identifying number (UIN)

1. Name of the registry: not applicable.
2. Unique identifying number or registration ID: not applicable.
3. Hyperlink to your specific registration (must be publicly accessible and will be checked): not applicable.

## Guarantor

Gizachew Gobebo Mekebo, E-mail: gizmake@gmail.com

## Data availability

All data used for this study are available upon reasonable request from the corresponding author.

## Provenance and peer review

Not commissioned, externally peer-reviewed.
